# 3D printed full-arch versus digital reference dental models: A systematic review

**DOI:** 10.6026/9732063002001100

**Published:** 2024-09-30

**Authors:** Gaurang Mistry, Charushila Sardar, Prerna Pandey, Mishal De Souza, Vibha Kailaje, Sanpreet Singh Sachdev

**Affiliations:** 1 Department of Prosthodontics, D.Y. Patil Deemed to be University, School of Dentistry, Navi Mumbai, Maharashtra, India; 2 Department of Oral Pathology and Microbiology, Bharati Vidyapeeth (Deemed to be University) Dental College and Hospital, Navi Mumbai, Maharashtra, India

**Keywords:** 3D Printing, Dental Models, Accuracy, Digital Light Processing

## Abstract

The present systematic review and meta-analysis aimed to evaluate and compare the accuracy of different 3D printing techniques used for fabricating full-arch dental models against digital reference models. The review included studies that assessed the accuracy of stereolithography (SLA), digital light processing (DLP), PolyJet and fused filament fabrication (FFF) technologies. A total of seven studies were analyzed, providing insights into the trueness and precision of 3D-printed models. The findings reveal that while all examined 3D printing technologies produced models with clinically acceptable accuracy, DLP and PolyJet techniques consistently demonstrated superior precision and trueness compared to SLA and FFF. The results indicate that DLP and PolyJet technologies are particularly suitable for applications requiring high dimensional fidelity, such as in Prosthodontics. However, the studies also highlighted some limitations, including small sample sizes and variations in study design, which may impact the generalizability of the results. Future research should focus on large-scale clinical trials and explore the impact of post-processing on model accuracy. This review underscores the importance of selecting appropriate 3D printing technologies based on clinical requirements to ensure optimal outcomes in dental prosthetics.

## Background:

Additive manufacturing (AM), commonly known as 3D printing, involves the layer-by-layer deposition of materials to transform digital designs into physical objects. In the field of Dentistry, the adoption of 3D printing technology has grown significantly, finding applications in Prosthodontics, Orthodontics, Implantology, and Oral and maxillofacial surgery. A key application of this technology is the fabrication of dental models [1]. For dental models to be effective, they must accurately replicate the teeth and surrounding tissues, serving as crucial tools for diagnosis, treatment planning, and the creation of various dental prostheses. However, traditional cast models present several challenges, including the need for immediate processing of impressions, which varies based on the impression material used. Furthermore, they require considerable storage space and involve substantial human and laboratory resources [2]. On the other hand, 3D-printed models offer a more streamlined and resilient workflow, allowing for on-demand production that reduces both time and labor [3]. Still, there are some limitations. The accuracy of 3D printed models can be affected by several factors, such as data acquisition, image processing of the oral hard and soft tissues, and the many parameters involved in the manufacturing and post-processing stages [4]. Various 3D printing technologies are currently available, each utilizing different techniques and yielding varying levels of performance and output. This variability makes it challenging to establish a standardized measure of accuracy. The most widely used technologies include stereolithography, digital light processing, material jetting, and fused filament fabrication. Other methods like continuous liquid interface production and binder jetting are also in use but are less common [5]. Definitive dental casts can now be created using either subtractive or additive manufacturing technologies. Additive manufacturing also referred to as rapid prototyping or 3D printing, involves constructing objects layer by layer [6]. This technique has become integral to the digital workflow in dental restorations. The use of 3D printing spans various dental fields, including maxillofacial prosthetics [7], orthodontic treatment planning [8] as well as surgical and implant dentistry [9]. SLA printers use ultraviolet lasers to solidify photosensitive resin layers [10]. DLP printers, in contrast, employ high-powered LEDs and photosensitive resins, utilizing micromirrors that individually control light reflection to minimize build time [11]. Polyjet or material jet technologies involve extruding materials through nozzles or jetting a photopolymer across the workspace, which is then solidified using a UV light source [12]. The accuracy of 3D printed models is assessed for their trueness and precision. Trueness refers to how closely the printed model matches the actual dimensions of the original, while precision measures the consistency of dimensions across repeated prints. High trueness indicates that the printed object closely aligns with its intended dimensions, while high precision signifies that the 3D printer consistently produces objects with the same dimensions across multiple prints. Although there has been research on the accuracy of 3D printed objects, studies specifically focusing on the accuracy of 3D printed dental working models remain limited [13, 14-15]. The present systematic review, thus, is conducted to comparatively evaluate the accuracy of 3D printed full-arch dental models manufactured using different printing techniques with digital reference models.

## Methods:

A systematic review of literature and meta-analysis was performed. This study followed the (PRISMA-DTA) Preferred Reporting Items for Systematic Review and Meta-Analyses statement guidelines for diagnostic test accuracy studies, the Cochrane Handbook for systematic reviews of interventions, version 5.1.0. and 4th Edition of the JBI Reviewer's Manual and was registered at PROSPERO under registration code CRD42023473585 [16].

## Eligibility criteria:

## Inclusion criteria:

[1] Population - Studies including maxillary and mandibular full arch Dental models.

[2] Intervention - Studies including 3D printing technology for assessment of dental models.

[3] Comparison - Studies including A digital reference model, which is the initial virtual model of the object to be printed, is expressed in the standard tessellation language (STL) file format.

[4] Outcome - Studies giving information about accuracy of 3D printed models as compared to the digital reference models.

[5] Study design - Studies published in English language only.

a. Studies published between 1-1-2000 to 30-11-2023.

b. Study design used - *in vitro* studies, clinical trials, Randomized controlled trials, Non-RCTs or quasi experimental studies, Cross-sectional studies.

## Exclusion criteria:

[1] Reviews, case reports, case series and animal studies.

[2] Studies providing only abstract and not full text.

[3] Studies available in languages other than English

## Focused review question:

Is there any difference in the diagnostic accuracy of 3D printed full-arch dental models manufactured using different printing techniques with digital reference model?

## Search strategy:

Studies were selected based on the PICOS inclusion criteria in the review protocol. Two reviewers assessed titles and abstracts to identify potentially eligible studies. Any queries were discussed with a third reviewer.

[1] The preferred reporting Items for Systematic Reviews and Meta-Analyses (PRISMA) for conducting a meta-analysis were followed.

[2] The electronic data resources consulted for elaborate search were PubMed, DOAJ, EBSCO, k-hub and Google Scholar with controlled vocabulary and free text terms.

[3] Articles published from 01/01/2000 until 30/11/2023 were searched.

[4] Following keywords and MeSH terms were used in combination with Boolean operators in the advanced search option.

## Data extraction:

Two reviewers independently extracted data from the included studies. Disagreements were again resolved through discussion. Authors, Year and Title of study, Country, Sample size, Study design, 3D printing method, Reference scanner, 3D analysis software, Outcomes, Results and other items were recorded. Data extraction was done and accurately recorded in the Excel sheets for all the primary outcomes separately.

## Critical appraisal of retrieved studies:

Quality Assessment of the selected studies was performed using the QUADAS-2 tool which included key domains - patient selection, index test, reference standard, flow, and timing.

## Results:

## Study selection:

**Figure 1:**
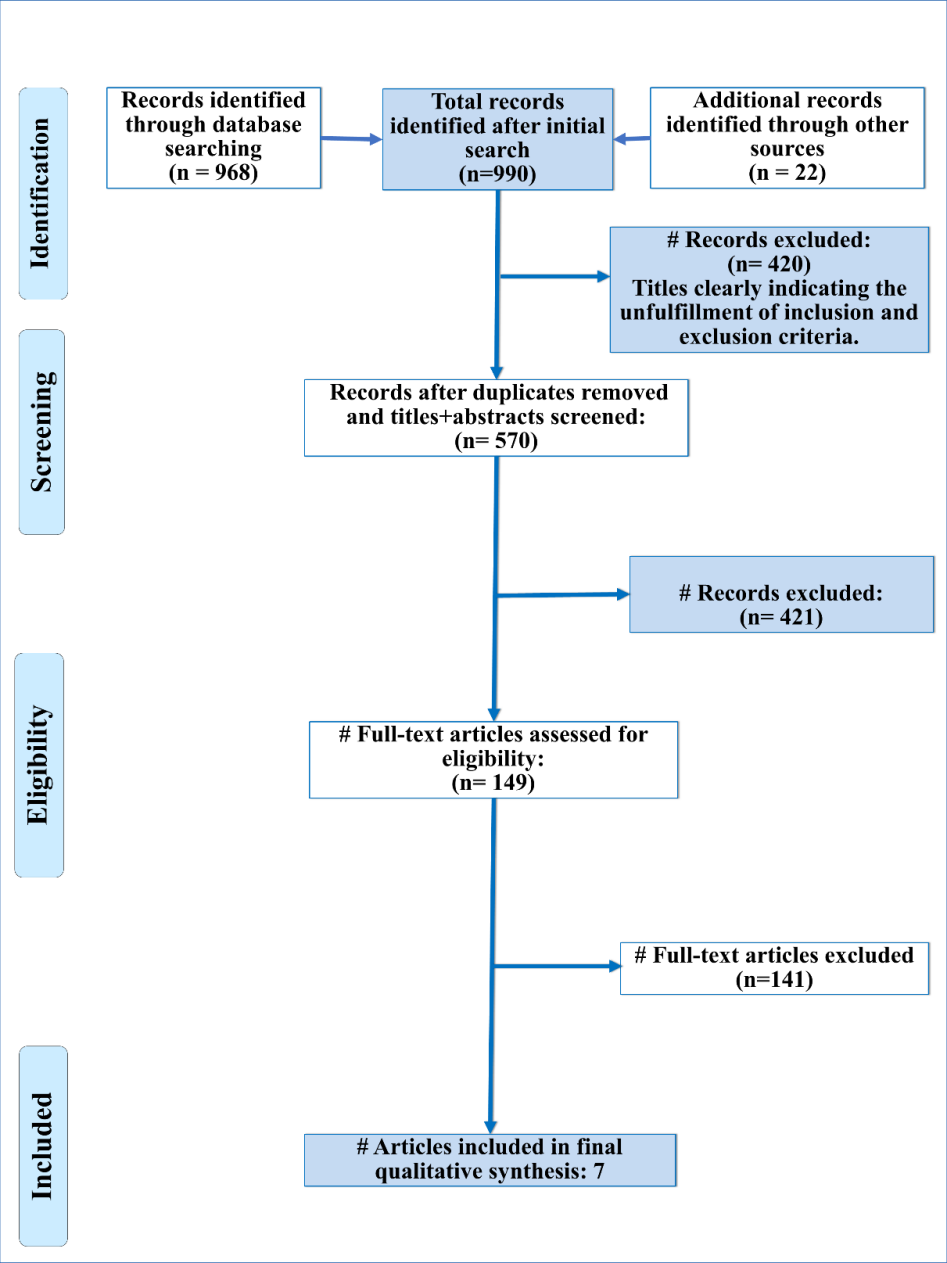
PRISMA flow diagram

Seven studies were included in the qualitative synthesis which was subjected to data extraction and quality assessment [17, 18, 19, 20, 21, 22- 23]. (Figure 1). The general characteristics are summarized in Table 1. All the studies showed *in vitro* study design assessing the accuracy of 3D printing methods with digital reference models. The included studies were conducted in different parts of the world such as Romania, Korea, Turkey, Boston, Russia and Italy. The conclusions of all studies stated that the 3D printed models showed acceptable accuracy as compared to the digital reference models. Among the different 3D printers used, DLP showed more dimensional accuracy with the reference models.

## Risk of bias assessment:

**Figure 2:**
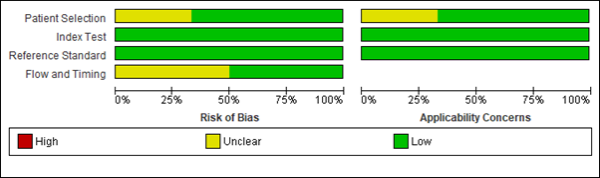
Risk of bias graph

**Figure 3:**
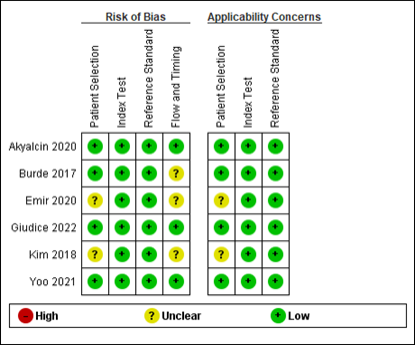
Risk of bias summary

Among the included studies, two showed moderate risk of bias and remaining five studies showed low risk of bias. (Figure 2 and Figure 3) (Table 2). In study by Emir 2020 and Kim 2018, information related to patient selection (in this case model selection) was unclear which raised the applicability concerns. Also, information pertaining to flow and timing was inadequate. This led to moderate risk of bias in these studies.

## Discussion:

The findings of this systematic review and meta-analysis highlight the critical role that 3D printing technology plays in the fabrication of full-arch dental models. The study aimed to compare the accuracy of different 3D printing techniques - specifically, SLA, DLP, PolyJet and FFF- against digital reference models. The results indicate that while all 3D printing technologies assessed in the included studies generally produced models with clinically acceptable levels of accuracy, there were notable differences in trueness and precision among the different techniques. The review revealed that DLP and PolyJet technologies consistently produced the most accurate models when compared to other 3D printing techniques. These findings align with the literature, which suggests that DLP and PolyJet methods are superior due to their high resolution and precision in layer-by-layer material deposition [24]. The higher accuracy of these methods makes them particularly suitable for applications where dimensional fidelity is critical, such as in the creation of working models for fixed prosthodontics. SLA, though producing clinically acceptable models, showed slightly lower accuracy compared to DLP and PolyJet. This discrepancy could be attributed to the differences in the photopolymerization process and the resolution of the printing equipment. The study by Kim *et al.* (2018) and Emir *et al.* (2020) further supports this, demonstrating that even though SLA is a reliable option, DLP and PolyJet methods provide enhanced precision, especially in intricate dental structures [18]. From a clinical perspective, the findings suggest that while all evaluated 3D printing techniques are viable for producing dental models, the choice of technology should be guided by the specific clinical requirements. For instance, in scenarios where maximum accuracy is paramount, such as in the fabrication of crowns, bridges, or implant-supported prostheses, DLP or PolyJet printers might be the preferred choice. However, for applications where slight variations in model accuracy are tolerable, such as in orthodontic study models or preliminary diagnostic tools, SLA and FFF printers may offer a cost-effective alternative [8]. Despite the valuable insights provided by the included studies, several limitations were noted. The majority of the studies had small sample sizes, which may affect the generalizability of the results. Additionally, the studies were primarily *in vitro*, limiting the applicability of the findings to real-world clinical scenarios. The heterogeneity in study designs, printing parameters, and reference scanners used across studies also presents challenges in drawing definitive conclusions. The moderate risk of bias identified in two studies (Emir 2020 and Kim 2018) further underscores the need for caution when interpreting the results [18, 19]. To build on the findings of this review, future research should focus on conducting large-scale clinical trials that assess the accuracy of 3D printed models in vivo. This would provide a more comprehensive understanding of how different 3D printing techniques perform under clinical conditions. Additionally, studies exploring the long-term dimensional stability of 3D printed models and the impact of post-processing procedures on accuracy would be beneficial. As 3D printing technology continues to evolve, it is crucial to continually reassess the accuracy and clinical utility of these methods to ensure optimal patient outcomes.

Overall, findings from this systematic review and meta-analysis indicated that while all evaluated 3D printing techniques can produce full-arch dental models with acceptable accuracy, DLP and PolyJet methods offer superior trueness and precision. Clinicians should consider these findings when selecting 3D printing technologies for dental model fabrication, balancing the need for accuracy with cost and material considerations. Further research is needed to validate these findings in clinical settings and to explore the potential of emerging 3D printing technologies in Dentistry.

## Conclusion:

The Comparative evaluation of accuracy of 3D printed full-arch dental models manufactured using different printing techniques with digital reference models depicted that Among the included studies, two showed moderate risk of bias and remaining five studies showed low risk of bias and it was observed that the 3D printed full arch dental models were more accurate compared to Digital Reference Model.

## Figures and Tables

**Table 1:** Characteristics of included studies

**Study ID**	**Place of study**	**Sample size**	**3D printing system usedReference standard**	**Model design**	**Reference scanner**	**3D analysis software**	**Outcomes assessed**	**Conclusion**
Burde 2017 [21]	Romania	20 each group	3DReshaper, Model Creator, Technodigit, Genay, FranceGrey light curing resin (GPGR02, Formlabs Gmbh, Berlin, Germany)	Mandibular and maxillary horse-shoe shaped model	InEos X5, Sirona Gmbh, Bensheim, Germany	Geomagic Qualify 13 (Geomagic, Morrisville,USA)	trueness	FDM models: more dimensionally accurate, less affected by mesh integrity
Kim 2018 [22]	Korea	5 each group	ZENITH (Dentis,Daegu, Korea) Mone (MAKEX Technol-ogy, Zhejiang, China) Cubicon 3DP-110F (HyVISION System, Sungnam City, Korea) Objet Eden 260VS; Stratasys, Eden Prairie, Minndigital reference model	pair of typodont horse-shoe shaped models with half-ball markers	Identica Hybrid (MEDIT, Seoul, Korea)	Geomagic Control (3D Systems, Rock Hill, SC)	precision, trueness	Significant differences: PolyJet and DLP more precise than FFF and SLA, PolyJet highest accuracy
Emir 2020 [23]	Turkey	10 each group	RapidForm XOR2, 3D Systems Inc., USAstereolithography (SLA) system	An arch-shaped master model to simulate the mandibular arch (14 mm in height and 16 mm in width)	blue LED light 3D scanner (ATOS Core 200 5M, GOM GmbH, Braunschweig, Germany)	Geomagic Control, 3D Systems	precision, trueness	Significant differences: DLP more accurate, all models within clinical tolerance, clinically acceptable for fixed restorations
Akyalcin 2020 [24]	Boston	20 each group	M2 Printer (Carbon) Juell 3D Flash OC (Park Dental Research, NY) Form2 (Formlabs Inc.,Somerville, Mass) Objet Eden 260VS (Stratasys, Eden Prairie, Minnraw images in .STL format converted using Dolphin Imaging and Management Solutions	horse-shoe shaped maxillary and mandibular dental arch models	iTero Element intraoral scanner (Align Technology,Santa Clara, Calif).	Geomagic Control (version 2015.3.1, 3D Systems, Rock Hill, SC, USA)	trueness	Surface area: not identical to original scan data, affected by printer type
Mangano 2020 [25]	Russia	3 each group	(Shera, Lemforde, Germany) Solflex350 (Voco, Cuxhaven, Germany) Form 2 (Formlabs, Somerville MA, USA) Vida HD (Envisiontec, Gladbeck, Germany) XFAB 2000 (DWS Systems, Thiene, Vicenzam Italy) MOONRAY D75 (Sprintray Inc., LA, CA, USA)digital reference model	horse-shoe shaped maxillary model	Freedom UHD desktop scanner	engineering software program (Studio 2012)	trueness	Acceptable accuracy, statistically significant differences among models
Giudice 2022 [26]	Italy	N/A	Elegoo Mars Pro (Shenzhen Elegoo Technology Co., Shenzhen, China) and the Anycubic Photon S (Anycubic Technology Co., Shenzhen, China).digital reference model	maxillary dental typodont	T710 desktop scanner (MEDIT, Seoul, Korea)	3-Matic research software (vr. 13.0.0.188, Materialise, Leuven, Belgium)	trueness and precision	Entry-level LCD-based printers: less accurate than professional-grade, close to orthodontic clinical threshold values
Yoo 2021 [27]	Korea	12 per group	3D systemreference STL file	maxillary molar and premolars	industrial 3D scanner (E4 lab scanner, 3Shape, Copenhagen, Denmark)	Geomagic Control, 3D Systems	trueness and precision	DLP, MJP, and SLA models: clinically acceptable for manufacturing dental prostheses

**Table 2:** Quality assessment according to QUADAS-2 tool

**Study Id**	**Patient selection**	**Index test**	**Reference standard**	**Flow and timing**	**Applicability concern**	**Risk of bias**
Burde 2017	Low	Low	Low	Unclear	Low	Low
Kim 2018	Unclear	Low	Low	Unclear	Low	Moderate
Emir 2020	Unclear	Low	Low	Unclear	Low	Moderate
Akyalcin 2020	Low	Low	Low	Low	Low	Low
Mangano 2020	Low	Low	Low	Low	Low	Low
Giudice 2022	Low	Low	Low	Low	Low	Low
Yoo 2021	Low	Low	Low	Low	Low	Low
